# Use of Stimulant Substances Among University Students in Tehran: a Qualitative Study

**Published:** 2011

**Authors:** Afarin Rahimi-Movaghar, Golara Khastoo, Mahdieh Moinolghorabaei, Masud Yunesian, Ahmad-Reza Sadeghi

**Affiliations:** 1 Department for Mental Health and Substance Use Iranian Research Center for HIV/AIDS (IRCHA), Tehran University of Medical Sciences, Iran.; 2 Iranian Research Center for HIV/AIDS, Tehran University of Medical Sciences, Iran.; 3 Department of Psychiatry, Tehran University of Medical Sciences, Iran.; 4 Center for Environmental Research & School of Public Health, Tehran University of Medical Sciences, Iran.; 5 Tehran University of Medical Sciences, Iran

**Keywords:** Ecstasy, Iran, Methylphenidate, Stimulants, University Students

## Abstract

**Objective:** There is evidence to show an increase in use of stimulant substances among university students. This study is a qualitative assessment of the existing situation and the underlying reasons for stimulant use among the students of Tehran University of Medical Sciences in 2006.

**Methods:** Three qualitative methods have been used: focus group discussions with 7 groups (60 individuals) consisting of male and female students in the dormitories and in the university environment, in-depth interview with 20 drug user students, and interview with 20 key informants including counselors, managers and guards of dormitories, staff of the office for Culture and Welfare Affairs of students and members of students' organizations. Purposeful or opportunistic method was used for sampling.

**Results:** Ecstasy and methylphenidate (Ritalin) were mentioned as the stimulants used by the students. The main declared reasons for ecstasy use were desire to have fun and excitement, desire to modernity, participation in parties, curiosity and living without family. The high expense of ecstasy pills and the training provided by media were mentioned as the main protective factors. Most respondents believed that use of methylphenidate was more prevalent than ecstasy use. In contrary to the drugs used for fun and enjoyment, methylphenidate is used for increasing learning abilities and academic achievement. Other pointed- out factors were ease of use and being stigma-free.

**Conclusion** Increasing risk perception via training, social alternatives to risky activities and parental training for continuing care and advice in the college period are recommended.

## Introduction

Stimulant use is considered as a global phenomenon. According to the estimation of UNODC in 2008, approximately 34 million individuals, namely, 0.6 % of the 15-64- year-old population of the world has consumed a stimulant during the most recent year. This rate exceeds the rate of heroin or cocaine use. More than a half of the users reside in Asia. Stimulant use has been controlled in developed countries e.g; the United States, Canada, Britain, and the Western Europe countries while the rate is on the rise in developing countries, especially the Middle East (1).

3, 4-methylene- dioxy- metamphetamine (MDMA or Ecstasy) is a hallucinogen stimulant derived from amphetamine. This substance has been subject to the attention of the adolescents and the youngsters during the recent years. For years the use ecstasy was considered harmless; but today the extensive conducted research has shed light on its short- and long- term adverse effects. Some of the acute adverse effects of ecstasy include restlessness, anxiety, hyperthermia, hyponatremia, and brain edema. Use of MDMA causes a rocketing rise in the frequency of high-risk behavior and the consumers become accident-prone. On the other hand, the existing impurities of the substance can be counted as an underlying cause for hepatic and cardiac complications. The long-term adverse effects resulting from a decline in serotonergic activity include memory impairment, impaired judgment, lack of impulse control, and disrupted sleep- wake cycles (2-5). Methylphenidate (MP) is an amphetamine derivative and is mainly prescribed for the purpose of treatment of hyperactivity disorder in children or treatment- resistant depressive disorders. It is abused by some youngsters with the aim of increasing concentration. There are some brand names of MP mostly Ritalin^®^ and Stimdate^®^ in Iran. 

While the Netherlands is the first main production site for ecstasy; Belgium and Germany stand in the second place. Nevertheless, it is produced in other European countries as well as South- East Asia. It is estimated that 8 million capita have used ecstasy all over the world during the most recent year. The highest rate is reported from the East and South- East Asia while Europe, Australia, and the United States are reported to be the next consumption sites. The annual production rate is estimated to be 100-125 tones. Attention should be drawn to the fact that all over the world, ecstasy is abused by the youngsters attending dance clubs who use the substance for fun (6). 

In any community, university students represent the well- educated class, and their mental health status can be deemed as a representative of the future landscape of the society. This group lies among the very important groups in assessment of substance- related issues; to the extent that it is called “Monitoring the Future” group in the United States. Evidence provided in different areas of the world depicts the fact that ecstasy use is more prevalent among university students. Majority of the youngsters go through their first experience of independent life as they find their way to university; they step into the new culture and the norms of their comrades and form their own new social norms (7). Consequently, a more detailed knowledge of their behavior and the factors influencing it is of paramount importance. 

Throughout the past three decades, substance use has increased in Iran significantly (8). Lots of studies have been conducted on university students to assess their substance use (9); however a comprehensive search of the existing resources does not provide any qualitative study on stimulant use among university students. 

This paper presents a part of a more extensive study, which aims at studying the status of substance use and abuse, recognition of the factors contributing to start and continuation of substance use, rate and routes of access to substances among students enrolled in Tehran University of medical sciences in 2006. The University is the eldest one in the country, where more than 4000 students are studying in. The ultimate objective of the study is to provide practical recommendations for design of substance abuse prevention interventions for university students. This paper evaluates the status and the factors influencing stimulant use including ecstasy and methylphenidate among students of Tehran University of Medical Sciences. 

## Materials and Methods

The present study has been conducted in Tehran University of Medical Sciences. Three parallel methods have been utilized: Focus Group Discussions (FGDs) with different subgroups of students, in- depth interviews with drug user students, and interviews with the key informants. 

Focus Group Discussions were conducted for seven groups of students, which included 60 individuals: two female and two male student groups residing in university dormitories participated in FGDs, while three other focus group discussions (two male and one female group) took place in the university environment. The list of the dormitories of the University was received from the Deputy of Student Affairs. The deputy did not have access to any information regarding the pensions where students lived. With respect to the fact that students residing in private flats did not welcome the research team inside their residence, samples of these students were placed into FGDs along with students living in Tehran. Overall, the necessity of conducting two sessions each lasting one hour and a half with the same participants and the interference with the time of their lectures and examinations, conduction of the FGDs was a difficult task to perform. 

In-depth interviews were conducted with 20 drug user students. Several approaches were used to get access to these students. These approaches included: requesting university counseling centers, the managements of dormitories, installation of advertising sheets in the faculties and the dormitories of the university providing the information that students with any experience of substance use would receive book coupons in case they made a telephone contact to provide information required by the research team. The other pathway to get access to information was asking individuals about any history of substance use in case observations inside the university and the appearance of the individual were suggestive of substance use. 

Twenty key informants were also selected to be interviewed. In consultation with the management of dormitories’ affairs and the management of student relations, the key informants were selected. While conducting other interviews, other key informants were recognized and introduced to the team. These mainly included counselors, managers of students’ affairs, direct managers of the dormitories, the security guards serving in different shifts in the dormitories and the members of students’ organization. 

Based on the qualitative nature of the study, the sample was selected through opportunistic or purposeful method, and random sampling was not considered as the method of choice. The research team tried to include individuals representing different groups of the population (considering gender and the faculty); the same methods were applied for selection of key informants. Researcher- structured questionnaires were used for the three groups; the questionnaires mainly consisted of open questions, which were accompanied with some semi- structured questions. 

The field work was performed in 2006. The interviewers’ group consisted of physicians and psychologists experienced in drug abuse treatment and were trained on using the questionnaires. All the interviews were performed with close supervision of the main research team members so that data collection process was homogenous as far as possible while flexibility was also considered in the process. The qualitative data provided by the in-depth interviews and the FGDs were extracted manually and analyzed through content analysis method. In the first step, the completed questionnaires were reviewed by the research team, and then the key words and concepts were extracted based on which the report was classified. Eventually, the responses were summed up and the results were analyzed and interpreted. 

In order to keep in lines with ethical codes, the interviews were based on voluntarily participation and all the participants were included after informed consent. The questionnaires did not register the names of the participants and the research team kept the completed questionnaires as confidential and only the sum up of the data was presented in the reports. It should be noted that due to the illegal and sensitive nature of the subject of the study, provision of information by the participants was accompanied with some resistance. 

## Results

*Characteristics of the interviewees*

The 20 key informants included two counselors, five managers of students’ affairs office, seven direct managers of the male and female university dormitory complexes, four dormitory security guards working in different shifts, and two from the students’ association). Twelve (60%) of the key informants were male and the rest were female. Their age ranged from 24 to 53 years (mean=35), and their work experience in university ranged from two to 30 years (mean=12). Their education ranged from junior school to PhD level, with participants holding a bachelor degree having the highest frequency. Despite their lower education, the old security guards were found to be the most informed interviewees. 

Of the 20 substance user students who were subject to in-depth interviews, 15 (75%) were male. Their age ranged from 19 to 24 years (mean=21). More than 70% were in their third or fourth year and the rest (30%) were in either the first or the second year of university education. These participants were from different disciplines of medical university, namely, dentistry, medicine, pharmacology, radiology, environmental health, nursing, and speech therapy. Forty percent were essentially from Tehran and the rest came from other districts of the country. 

Seven groups of students (including 60 interviewees) were arranged for FGDs. Their age varied from 19 to 24 (mean=22), and were studying in 14 different majors at different levels. Thirty percent were from Tehran while others came from other districts. 

*Stimulants*

It is essential to notify that when asked about stimulants, the respondents generally interpreted “stimulant” as ecstasy among university students. Since the participants did not automatically recognize Ritalin (methylphenidate) as an illegal substance causing addiction, they were all asked about methylphenidate specifically and separately. 

*Prevalence of ecstasy use*

About 50% of the key informants, 50% of the substance user students, and 80% of the students participating in FGDs declared that they were unaware of the experience of ecstasy use among university students. 

While the informed key individuals believed that 5% of the male and 3% of the female students experienced use of ecstasy or the “Joy” tablets, majority of the informed students estimated these figures to be 10 and 5%, respectively. The key informants also considered 1% of the male and 0.5% of the female students to be constant users of ecstasy. Majority of the students believed the figures were 5 % for the male and 2% for the female students. Overall, students estimated higher rates for ecstasy use compared to the key informants. 

Some of substance user students believed that the use of such substances had become more frequent during the past one or two years (years 2004 and 2005). Based on their report, the prevalence of ecstasy use had decreased to some extent at the time. One of the substance user students noted: “*Ecstasy use is more frequently observed among rich male students from Tehran who are keen on participating in parties. Up to 10% of this group might have been through the experience although there has been a significant decline in the rate of ecstasy use as a result of the efforts of the mass media to raise public awareness on the hazards of the substance*.” 

One of the female students participating in FGDs pointed out: “*I had been informed by friends participating in parties that the rate of use was higher among girls as compared to boys, and up to 30% of the girls who showed up in parties used ecstasy*.” 

One of the female students participating in FGDs pointed out: “*I had been informed by friends participating in parties that the rate of use was higher among girls as compared to boys, and up to 30% of the girls who showed up in parties used ecstasy*.” 

*Factors contributing to ecstasy use*

Based on the information provided by the three respondent groups (key informants, substance user students, and students participating in FGDs), *individual and family factors* contributing to ecstasy use include:

- Tendency to experience joy, having fun, increase energy and the breezy nature of ecstasy;
- Curiosity for novel sensory and emotional experiences;
- Tendency to have adventures and taking risks;
- The concept of modernity of ecstasy use and tendency to newfangled behavior;
- Interest in participating in parties and the availability of the substance in parties;
- Pretending bravery, avoiding counted as “coward” by others, especially by the opposite sex;
- Considering prohibited and illegal leisure as a value;
- Unattractive nature of other pastimes;
- Having a good economic status;
- Lack of control and supervision of the family, and living with friends as singles.

The most important and frequent underlying reasons for ecstasy use were mentioned to be “tendency to experience joy” and “the concept of modernity of use of the substance”. One of the substance user students declared: “*Use of the substance is most frequently seen among kids imitating rich people as if they belonged to higher economic class of the society*.” Another peer added: “*Ecstasy use is more frequently observed among students with a better economic status, especially because of the positive phase, the freshness feeling, and the laugh it is followed by; especially by Tehrani boys who tend to participate in parties*.” 

The respondents reported the factors related to *the university and the student life environment* as below:

- Arranging weekend parties in homes of Tehrani students and high prevalence of ecstasy use in these parties;
- Lack of any odor as compared to alcoholic beverages, cannabis, and as a result, lower risks at the time of return to the dormitory;
- Tendency to become a part of a homogenous group of the university students in these parties;
- The concept of obscenity of use of other substances, such as heroin, and considering ecstasy use a high-class habit among university students.

It was noted that even some of the female students tended to over-present their own experience and were proud of stating their experience in front of others. Some mentioned that the use of ecstasy was more prevalent among students of the majors in which there was less academic pressure and among those who had more frequent get-togethers. 

The respondents believed that the *extrinsic factors outside the university environment* included:

- Changes in the norms, or abnormality of the lives of the youngsters and consideration of the newfangled behaviors as behavioral models;
- Rapid cultural revolutions, liberalism of the youngsters and their tendency to go through ANY experience,
- Interaction with other users and considering them as role models;
- Social pressures resulting to integration of the individual in the communities where any behavior is welcome;
- Lack or inadequacy of the appropriate leisure proportionate to the excitement needed by youth;
- Attending non- student parties by those who want to avoid their leisure being disclosed in the university or the dorm;
- Variety of the substances available in the market and their accordance with the adventurous nature of some of the students.

Majority of the respondents believed in the positive influence of the educational and awareness- raising programs broadcasted by the mass media throughout the most recent year; these programs eventually resulted in a more cautious attitude. The higher cost of ecstasy as compared to other substances available in the market was also highlighted as a limiting factor. One of the substance user students mentioned: “*I experienced ecstasy once or twice in the parties of rich people; either fortunately or unfortunately, I could not afford it thereafter!*” 

Based on the viewpoints of the respondents, the most significant factors in continuance of ecstasy use included gaining desirable results from the very first experiences, e.g.; having more fun and being invited to subsequent parties. One of the students noted: “*The guys who do not join the peers in their substance use will never be invited again*.” 

*Methylphenidate Use*

All participants knew and used the term Ritalin instead of the generic name of methylphenidate (MP). In this article, we use the term MP, as other brands of MP might be used and abused as well. About 50% of the key informants, 10% of the substance user students and 30% of the students participating in FGDs declared their unawareness of the experience of MP use. Majority of the key informants believed that the rate of MP use by students exceeded ecstasy use, to a great extent. 

The most frequent answer about the rate of MP use among male and female students was 20% and 10%, respectively. Meanwhile, the rate of constant use of MP was believed to be 5% among males and 2% among females. Majority of the respondents believed that as MP was used as a medicine, students did not find its use as a wrong habit. 

*Individual factors* contributing to MP use were mentioned as more positive attitude towards it as compared to other abusive substances (especially among students of Medicine), more curiosity around the effects of its use, academic competition of the students, and the belief that MP would never cause dependence. 

Respondents believed that the factors related to *the university environment and student life- style* influencing MP use mainly included its more normality in the students' culture, which in turn affected individual beliefs and values. Sometimes even those in their internship period advised the junior students to use MP. Lack of objective-oriented planning, use of MP for enhancement of learning at the final exams’ season instead of having a well-structured program for studying during the trimesters and the greed of the youth to have an easy student life were mentioned as other contributing factors. Majority of the respondents believed that the rate of MP use increased during the exams’ season. It was also mentioned that the students of Medicine has easier access to the substance to the extent that they could provide it for themselves and their friends. It was mentioned repeatedly that most of the users either were studying in one of the medial majors or had been encouraged to use the substance by people who had the authority to prescribe it and this was the main reason for regarding MP use as a normal behavior. 

One of the substance-user students noted: “*Ritalin use is more frequent among senior students, especially students of Medicine during exams’ season and their shift nights. They say it has been prescribed by their physician. Seven to eight students of the 2003-2004 have honestly told me in person that they use Ritalin during the exams’ season. However, through discussions with friends who attend other majors such as art and technology, we are aware that Ritalin use is even more prevalent among these students; at least 30% of them use Ritalin.*” 

Respondents believed that the most important *extrinsic factors* regarding MP use included easy access to the substance, and lack of recognition of its use as an obscene habit causing addiction by the public. Majority of the respondents believed that MP use would not cause legal problems for the individual. 

## Discussion

Substance use is a phenomenon leading to various physical, mental, social and economic harms, sometimes to the extent that the individual experiences serious decline in his/her individual or social function. Substance use is also considered as a contagious phenomenon, which endangers the peers and friends of the substance-user individuals. One study has depicted the fact that an ecstasy user will only need 15 months to make half of his/ her friends to use it (10). Therefore, this fact requires serious but at the same time scientific act of the responsible bodies. Evaluation of the different aspects of the phenomenon is deemed quite necessary in order to plan an appropriate response. Qualitative studies can be considered as one of the steps based on which future response is planned. Due to their qualitative nature, these studies can be counted as the supplement for the quantitative studies. The possibility of rapid conducting of such researches, their low costs, and the extended data provided, the qualitative studies are considered as effective tools for managers and program planners. In majority of the cases, these studies increase both reliability and validity of the results by means of multiple data collection methods that is called *triangulation*. 

In the present qualitative study, majority of the respondents estimated the rate of the experience of ecstasy among male and female students to be 5-10% and 3-5%, respectively. Constant use of ecstasy was reported to be rare. Even though quantitative studies are the preferred method for assessing the prevalence of the phenomena, it is recommended that the accuracy of self-reports in surveys, regarding prevalence of substance use are confirmed through qualitative studies and estimations provided by the key informants about the population being studied. Although the estimations might be influenced by several biases, but these can be diminished by including several types of well-informed groups of respondents, such as the three groups included in present study. 

Throughout the past decade, there has been a significant increase in the produced and published science regarding the nature and the extent of substance use in Iran. In the same ground, a significant number of epidemiologic studies have addressed substance use and use disorders among university students. These studies have been all reviewed in a systematic review (9), which provides the summation of 17 studies conducted throughout 1997- 2003. The systematic review has concluded that alcohol use must be more prevalent than the other assessed substances among university students. Opium and Cannabis ranked second and third. The review retrieved only three studies on stimulant use among university students. In 1999, lifetime prevalence has been reported to be 0.4 % in female students in Tehran. In 2001, a study in Mashhad reported a lifetime prevalence of stimulants use of 1.4%, and daily use of the substance of 0.2 % without any differentiation for the two genders. In another study conducted in 15 cities in 2002-2003, the results have not been reported separately for the two genders, either. The study reported a lifetime prevalence of 0.4% of ecstasy use. 

Moreover, Iranian researchers have been more focused on this issue during the most recent years. A study on lifetime prevalence of ecstasy use in six universities in Tehran during the academic year of 2005-2006 it as 0.7% (11). At the same time, the lifetime prevalence of stimulant use among male university students in Rasht city has been reported to be 7.25% (12). In 2004, a study on university students of Birjand city found a very limited awareness of the students on ecstasy and a lifetime prevalence of 4.3% (13). Moreover, some studies have addressed the young population, not considering whether the sample members were university students or not. In 2004, lifetime prevalence of ecstasy use among youth in the age range of 15-25 attending the coffee-shops of five different regions of Tehran was 18.5% (14). The same indicator was reported to be 7.6% (15) among the adolescents in the range of 16-18 years in the west of Tehran. Another study including high school students of Lahijan district in 2005 points out the prevalence of ecstasy use to be 2.4% (16). In two other studies the rate of ecstasy use by high school students of Gonabad and Rasht, was significantly lower, namely less than 1% (17). In general, the spectrum of the figures reported for ecstasy use is quite wide and at least among university students it ranges from 0.4% to 7.25%. The estimated rates provided by our study seem to be close to the upper extreme of this spectrum. 

In the United States, assessment of ecstasy use has been integrated into the national longitudinal study since 1989. Between 1989 and 1994, ecstasy use had a low prevalence rate but through 1994 to 1997, the last year use increased from 0.5% to 2.5%; this rocketed to 9.2% by 2001. At the point of 2003, there seems to be a turning point since the direction of the graph reverses and the prevalence rate declines to 2.2% in 2007. Researchers link the decline in ecstasy use with the very extensive education throughout the mass media and the raised awareness of the different subgroups of the community regarding the adverse effects of ecstasy. Such changes in use of ecstasy have been observed not only among university students but also among high school students as well as the youngsters not attending university (18). 

In Britain and Australia, stimulants use including ecstasy use rank second after cannabis use among all illegal substances (19, 20). In a study conducted on students of Medicine in Turkey, 4% reported a history of illegal substance use in their lifetime. These substances mainly included cannabis, ecstasy, and cocaine (21). 

In the present qualitative study, which has been conducted through in-depth interviews, and FGDs, several factors were considered as contributors to the use of ecstasy. These mainly included the breezy nature of ecstasy, the concept of modernity of use of ecstasy, participation in parties and living with single friends, curiosity and tendency to adventures, lack of any odor of the substance resulting in secrecy of the use, and lack of obscenity. On the other hand, the high costs of ecstasy pills and the education provided through the mass media have been named as the limiting factors for its use. 

Some of the factors contributing to ecstasy use have also been evaluated in cross- sectional quantitative studies. In one study conducted in six universities in Tehran, ecstasy use has been reported to be more prevalent among students not residing in dormitories rather than those in dormitories (11). In the study conducted on male university students in Rasht city (12) and the study on university students of Birjand city (13), the rate of ecstasy use was higher among university students residing in rental flats as singles as compared to those residing in dormitories or living with their parents. 

Among the youngsters in the range of 15 to 25 years referring to the coffee shops of five regions of Tehran, ecstasy use was significantly correlated with male gender, higher levels of income of the family, substance abuse and higher scores in Beck inventory (14). In the study conducted on a sample of adolescents in the range of 16 to 18 years in the west of Tehran, assessment of the correlated factors showed that almost half of these adolescents had a medium level of awareness regarding ecstasy and the adverse consequences of its use (15). In the study conducted on high school students of Lahijan district, the results of logistic regression showed that participation in peers’ parties, use of other substances and cigarette smoking were the factors correlated with ecstasy use. Among individuals participating in peers’ parties, lifetime prevalence of ecstasy use was 3.4 times more prevalent (16). 

On the other hand, evaluation of the most informed university students, namely, students of medicine in their internship in one of medical universities of Tehran in 2004 showed that the information of these students regarding ecstasy was not sufficient; one third of them were unaware of the potential of the substance to cause dependence. It might be interesting to note that only 17% of them had acquired their information regarding this substance through university courses (22). The results of another study conducted on general physicians of Kerman district illustrated that the information of a great number of the participants regarding ecstasy was quite inadequate (23). 

In a qualitative study in the United States through conduction of FGDs with 30 university students, majority of the respondents mentioned the positive effects of ecstasy on mood, sense of joy, mental changes and curiosity. They disclosed pressure of friends, easy access and easy use as the reason for ecstasy use (24). A cross-sectional quantitative study on the factors contributing to ecstasy use by American university students have illustrated the fact that ecstasy users are less religious and study less strictly. Meanwhile these individuals are more sociable, spend more time with their friends, and have more interests in arts (7). 

Regarding use of MP, majority of the participants of the present study estimated the prevalence rate to range between 10 and 20%. Most of them believed that use of this substance is more prevalent than ecstasy. In contrast to the high number of the studies conducted on substance use by university students throughout the recent decades in Iran, we did not encounter any Iranian study depicting the prevalence of the use of this substance separately and quantitatively. This shows that the use of this substance has not been a subject of interest for Iranian researchers. The reason might be the fact that this substance does not considered highly obscene. The other rational might be the belief of the researchers themselves that the substance use is accompanied with low harms and lacks abusive potentials. UNODC has reported that the prevalence of MP abuse is only significant in the United States and Australia (25). A systematic review showed that based on the 21 studies included in the review, the prevalence of last year use of MP among university students in the United States ranged from 5 to 35%. On the other hand, 16-29 percent of the university students with ADHD who had been prescribed MP, had mentioned a history of the sale of their medication (26). However, our qualitative study highlights the need for more studies on this substance. 

The present study also depicts the fact that in contrast to the other substances, which are mainly used for the purpose of joy and fun, MP is mainly used with the purpose of increased learning capacity and academic efficacy. Use of MP is accompanied with the incorrect brisk method of education and study in universities, namely, increasing memorized information at exams’ seasons. Use of MP is not accompanied with the sense of obscenity of use of other substances; this factor can be considered as a reinforcer for MP use. However, this fact is not limited to Iran. In a study conducted in 2006 in the United States, in-depth interviews with MP users shed light on the fact that its use took place mainly at the time of academic pressure. Decrease of fatigue, increase of learning abilities, it's easy and non- stigmatized use were mentioned as the main reasons for its use (27). Thus, it seems that the pattern and the contributing factors for MP use in Iran match the pattern found in the United States. 

Despite the increasing trend of stimulant use in some parts of the world, especially Asia, developed countries like the United States, Canada, and some of the European countries have succeeded to control and decrease the rate of stimulant use among their youngsters. In the United States, the rate of amphetamines’ use by high school students in 2006 has decreased to a third of the rate reported in 1997. Decrease of access to the substances and increase of the sense of harms lie among the most important reasons for this decline (18). 

Findings illustrate the fact that extensive and exact information provision regarding the hazards of the stimulants especially ecstasy and MP which are the most frequently used stimulants among university students, should be deemed as an essential requirement. Raising awareness through information provision must take place during adolescence and before stepping into university. Moreover, considering the influence of social beliefs especially the norms of the community of university students, preventive measures should be based on reinforcement of creative thinking and social skills of the individuals so that they become competent to act appropriately in different settings. 

One of the most important findings was the correlation of the use of these substances with peers’ parties; a fact that has also been notified by other studies conducted in other countries. Access to healthy and attractive social environments can be a substitute for risky leisure of the youngsters. It also seems that the care provided by parents should be continued in the age of university; supervision and presence of parents in peers’ parties is quite essential. 

## Notes

**Declaration of Interest:** None.
